# Deep hydroxyapatite deposition in porous poly(ethylene glycol) sponge hydrogel via optimized and simplified approach

**DOI:** 10.1080/14686996.2026.2620828

**Published:** 2026-01-23

**Authors:** Kaho Takada, Shohei Ishikawa, Rikima Kuwada, Lester Geonzon, Koichi Mayumi, Takamasa Sakai

**Affiliations:** a Department of Chemistry & Biotechnology, School of Engineering, The University of Tokyo, Bunkyo-ku, Japan; b Institute for Solid State Physics, The University of Tokyo, Kashiwa, Japan

**Keywords:** Hydrogel, hydroxyapatite, mineralization, poly(ethylene glycol), porous structure, phase separation

## Abstract

The controlled mineralization of hydrogels with hydroxyapatite (HAp) offers a promising route for engineering biomimetic scaffolds. However, conventional mineralization methods typically lead to surface-localized precipitation due to rapid ion depletion and limited transport within dense polymer networks, thereby restricting mineral penetration and compromising mechanical performance. Here, we present a simple sequential immersion protocol that achieves deep HAp deposition within a poly(ethylene glycol) (PEG) sponge hydrogel engineered via gel – gel phase separation and freeze – thaw processing. The resulting micron-scale porous architecture significantly enhances mass permeability, enabling bidirectional diffusion of phosphate and calcium ions. Structural and spectroscopic analyses confirm the formation of crystalline HAp throughout the hydrogel, while quantitative mapping of Liesegang ring patterns reveals extended mineral infiltration and nonlinear precipitation dynamics. Mechanical testing further demonstrates that mineralization reinforces the hydrogel without compromising its structure – overcoming the limitations of conventional alternating immersion methods. This work establishes a scalable and chemically straightforward strategy for constructing soft – mineral composites with tunable mineralization depth, advancing the design of bone-mimetic scaffolds and regenerative materials.

## IMPACT STATEMENT

Porous poly(ethylene glycol) sponge hydrogel enables simplified deep hydroxyapatite deposition, establishing a versatile synthetic platform for controlled mineralization and advancing hydrogel-based biomaterials design.

## GRAPHICAL ABSTRACT

**Figure:**
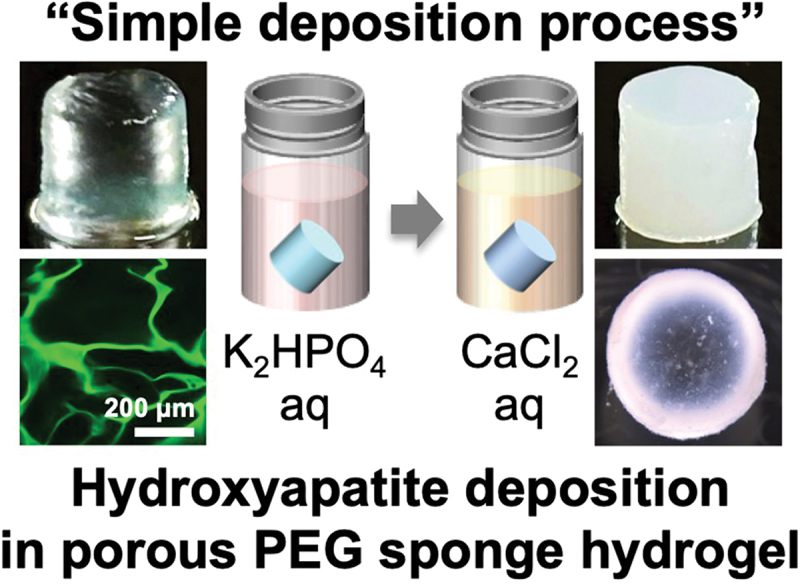

## 1. Introduction

Hydrogels – soft, water-rich polymer networks – have emerged as versatile platforms for engineering hybrid materials due to their tunable mechanical properties, chemical inertness, and compatibility with biologically relevant environments [1–3]. Among them, poly(ethylene glycol) (PEG)-based hydrogels stand out for their high biocompatibility, FDA approval, and ease of chemical functionalization [4–9]. These attributes have established PEG hydrogels as leading candidates in diverse fields ranging from tissue engineering to templated crystallization of inorganic phases [10,11]. A particularly compelling target for hybridization is hydroxyapatite (HAp; Ca_10_(PO_46_(OH)_2_), the primary mineral component of bone, which is valued for its osteoconductivity, biocompatibility, and chemical resemblance to native hard tissues [12–14].

In PEG systems, HAp mineralization is typically achieved through aqueous immersion protocols, leveraging the affinity between PEG chains and Ca^2+^ ions to promote in situ nucleation [10,12,15]. However, a persistent limitation in these systems is the restricted ionic transport and inefficient nucleation within dense hydrogel networks, which leads to shallow mineral penetration and surface-localized crystallization. This bottleneck not only compromises mechanical reinforcement but also impedes uniform functionalization of the bulk material. A striking phenomenological outcome of these constraints is the formation of Liesegang rings – periodic mineral bands that arise from nonlinear reaction – diffusion coupling between ions diffusing inward from the external solution and those outwardly diffusing from the hydrogel matrix [16]. While scientifically intriguing, these self-organized patterns underscore the limitations of ion diffusion and nucleation control, and present challenges for achieving deep mineral distribution in practical applications [17–19].

**Figure 1.**
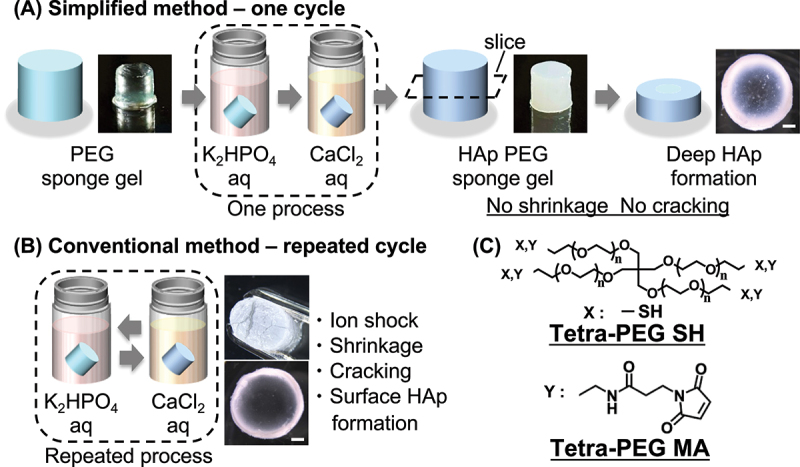
Preparation of hydroxyapatite (HAp)-mineralized PEG sponge hydrogels. (A) Schematic illustration of the optimized simple immersion protocol developed in this study. PEG sponge hydrogels were sequentially immersed in K_2_HPO_4_ and CaCl_2_ solutions under optimized conditions, resulting in uniform surface mineralization and deep mineral penetration throughout the hydrogel interior without macroscopic shrinkage. Scale bars, 1 mm. (B) Schematic illustration of the conventional alternating immersion method. Repeated immersion in K_2_HPO_4_ and CaCl_2_ solutions induces surface-localized mineralization, hydrogel shrinkage, and mechanical damage, as evidenced by surface cracking (top) and limited mineral penetration in the cross-sectional view (bottom). Scale bars, 1 mm. (C) Chemical structures of tetra-armed poly(ethylene glycol) (Tetra-PEG) functionalized with sulfhydryl (Tetra-PEG SH) and maleimide (Tetra-PEG MA) groups.

To address these challenges, we utilize a structurally engineered PEG sponge hydrogel as a new platform for deep mineralization. Developed via gel – gel phase separation (GGPS) followed by freeze – thaw processing, this hydrogel exhibits a highly porous, micron-scale interconnected architecture while being composed entirely of PEG [20,21]. Unlike conventional PEG hydrogels with nanometer-scale mesh sizes, the sponge architecture offers improved mass permeability without sacrificing the favorable Ca^2+^-coordinating properties of PEG chains. We hypothesized that this porous network would fundamentally alter the dynamics of mineral growth – enabling deep, bulk HAp deposition rather than surface-localized crystallization. To test this, we developed a simple, sequential immersion protocol designed to maximize ion penetration and minimize mechanical stress on the hydrogel. Unlike traditional alternating immersion methods, our approach induced crack-free, spatially deep mineralization that extended several millimeters into the hydrogel matrix (Figure 1). Crystallographic and spectroscopic analyses confirmed the formation of crystalline HAp throughout the hydrogel volume. Importantly, this was achieved without additives, porogens, or external stimuli, emphasizing the intrinsic advantages of the PEG sponge architecture. These findings highlight a powerful structural strategy for overcoming diffusion-limited mineralization in hydrogels. More broadly, this work redefines the interplay between hydrogel morphology, mass transport, and crystallization kinetics, offering a generalizable framework for engineering soft – mineral composites with controlled depth, spatial fidelity, and mechanical integrity.

## 2. Experimental

### 2.1. Materials

Maleimide-terminated tetra-armed poly(ethylene glycol) (PEG) and sulfhydryl-terminated tetra-armed PEG, each with a molecular weight (*M*_*w*_) of 10 kg/mol (referred to as Tetra-PEG MA and Tetra-PEG SH, respectively), were purchased from SINOPEG Biotech Co., Ltd. (Fujian, China). Alexa Fluor™ 488 C5 Maleimide (Alexa-MA) was obtained from Thermo Fisher Scientific Inc. (MA, U.S.A.). Tris(hydroxymethyl)aminomethane (Tris) was purchased from Sigma-Aldrich (MO, U.S.A.). Calcium chloride (CaCl_2_), citric acid, 99.8% deuterium oxide (D_2_O), dipotassium phosphate (K_2_HPO_4_), disodium hydrogen phosphate (Na_2_HPO_4_), Dulbecco’s PBS (D-PBS (-)), 6.0 M hydrochloric acid (HCl), sodium carbonate (Na_2_CO_3_), and sodium hydrogen carbonate (NaHCO_3_) were obtained from Wako Pure Chemical Industries, Ltd. (Osaka, Japan). Ultrapure water was prepared using a Milli-Q® SR 240 Purification System (Merck Millipore, MA, U.S.A.). The following instruments were used in this study: benchtop pH meter (LAQUA, HORIBA, Kyoto, Japan), confocal laser scanning microscope (CLSM, LSM 910, Carl Zeiss AG, Jena, Germany), compression tester (EZ Test EZ-SX, Shimadzu Corp., Kyoto, Japan), tensile tester (Autograph AG-X plus, Shimadzu Corp., Kyoto, Japan), optical microscope (M165C, Leica Camera AG, Wetzlar, Germany), ultrasonic cutter (ZO-40, Echo Tech, Chiba, Japan), and wide-angle X-ray scattering system (WAXS, Nanopix, Rigaku, Tokyo, Japan).

### 2.2. Solution preparation

Citrate phosphate buffer (CPB), Tris-HCl buffer, K_2_HPO_4_ solution, CaCl_2_ solution and carbonate-bicarbonate buffer (CBB) were prepared according to previously established protocols. The composition and pH condition were as follows: (i) CPB: 50 mM, pH 3.9 or 4.8. (ii) Tris-HCl buffer: 10 mM, pH 7.8. (iii) K_2_HPO_4_ solution: 30–600 mM K_2_HPO_4_ in Tris-HCl buffer. (iv) CaCl_2_ solution: 50–1000 mM CaCl_2_ in Tris-HCl buffer. (v) CBB: 10 mM, pH 9.75.

### 2.3. PEG hydrogel preparation

Tetra-PEG SH and Tetra-PEG MA were separately dissolved in CPB at optimal pH condition (PEG concentration (*C*_*PEG*_) = 10–20 g/L: pH 4.8, *C*_*PEG*_ = 30–60 g/L: pH 3.9). These two PEG solutions, prepared at identical concentrations, were mixed in stoichiometric balance and cast into a cylindrical polytetrafluoroethylene (PTFE) mold (diameter: 5.3 mm, height: 5.0 mm). The mixture was incubated at 25°C for 24 h to form Tetra-PEG hydrogel. To produce a sponge-like structure, the hydrogel was frozen at −20°C for 24 h, followed by incubation at 25°C for another 24 h. This hydrogel prepared via freeze-thaw process was defined as PEG sponge hydrogel. Unless otherwise specified, standard preparation condition was *C*_*PEG*_ = 60 g/L for PEG hydrogel and *C*_*PEG*_ = 10 g/L for PEG sponge hydrogel.

### 2.4. Hydrogel mineralization

Mineralization was performed via sequential immersion in four different solutions. Hydrogels were first immersed in the K_2_HPO_4_ solution (20–50 times hydrogel volume) at 25°C for 24 h, followed by washing with Tris-HCl buffer. The samples were then immersed in CaCl_2_ solution at equivalent volume to the K_2_HPO_4_ solution, maintaining a salt concentration ratio of [K_2_HPO_4_]: [CaCl_2_] = 3 : 5. After 3 h, the hydrogels were washed with Tris-HCl buffer and incubated at 25°C in CBB before being stored in D-PBS. For clarity, mineralization conditions using 300 mM K_2_HPO_4_ and 500 mM CaCl_2_ are denoted as K300 and C500, respectively. This notation, expressed as K or C followed by the salt concentration, is used throughout the manuscript. To assess the effectiveness of the designed mineralization method, we compared with a conventional alternating immersion protocol, in which the hydrogels were repeatedly transferred between K300 for 2 min and C500 for 2 min, with this cycle repeated 2 to 5 times.

### 2.5. Cross-sectional observation of mineralized hydrogels

Mineralized hydrogels were sectioned at the center using an ultrasonic cutter, and the cross-sections were observed under an optical microscope. Image analysis was performed using Image J to calculate the radial distribution of mineral deposition, based on the intensity of whiteness across the cross-sectional area.

### 2.6. CLSM observation

Fluorescently labeled PEG hydrogels were prepared by dissolving Alexa-MA (1 mg/mL) in DMSO and adding it to the Tetra-PEG SH solution (*C*_PEG_ = 10–60 g/L) at 0.5 vol%. Tetra-PEG MA was separately dissolved in CPB at the same concentration. The two PEG solutions were then mixed at a stoichiometric ratio, cast into cylindrical molds, and subjected to a freeze – thaw process. The internal microstructure of the resulting hydrogels was visualized using CLSM. Porosity and condensation ratio was analyzed using ImageJ software.

### 2.7. ^1^H T_2_ relaxation measurements

Hydrogels were immersed in D_2_O solution and incubated at 25°C for 24 h. After replacing the solution twice with fresh D_2_O to ensure equilibration, the ^1^H T_2_ relaxation measurements were performed at 25, 50 and 60°C using two pulsed sequences. A spin echo sequence was used to capture relaxation dynamics in the 0.1–20 ms range, while a Carr – Purcell – Meiboom – Gill (CPMG) sequence was employed for the 0.2–3500 ms range. The resulting relaxation decay curves were superimposed to generate a composite profile. The decay in signal intensity, *I*, was analyzed using a two-component exponential model:

$$I=A_{1}\left(\exp\left(-\left(\frac{t}{\tau_{1}}\right)\right)\right)+A_{2}\left(\exp\left(-{\left(\frac{t}{\tau_{2}}\right)}^{b}\right)\right)$$

Here, *τ*_1_ and *τ*_2_ represent the fast and slow relaxation times, respectively, and b is the stretching parameter for the slow component. *A_1_* and *A_2_* correspond to the relative fractions of the fast and slow components, such that *A_1_ + A_2_* equals the initial signal intensity.

### 2.8. WAXS measurement

The crystalline structures of mineralized hydrogels were analyzed using WAXS. Each sample was exposed for 300 seconds, with a detector-to-sample distance of 0.10 m. Silver behenate (AgBh) was used as the calibration standard. The acquired 2D WAXS images were converted into 1D scattering profiles. Data correction was performed in the following order: first, normalization by transmittance and exposure time; second, subtraction of background scattering from the empty cell; and finally, correction based on the sample thickness to ensure quantitative accuracy.

### 2.9. IR measurement

Hydrogels were placed on the IR measurement stage, and surface IR spectra were recorded using a JASCO FT/IR-4X spectrometer (JASCO Corporation, Tokyo, Japan). A single-reflection ATR unit equipped with a diamond prism was used.

### 2.10. Degree of swelling

To evaluate the swelling behavior following mineralization, hydrogels were immersed in solutions with varying concentrations of K_2_HPO_4_ (K) and CaCl_2_ (C). The initial diameter of the as-prepared hydrogel (*d_0_*) was measured prior to mineralization, and the post-mineralization diameter (*d*) was determined by microscopic observation. The equilibrium degree of swelling (*Q*) was calculated using the formula: *Q* = (*d*/*d*_0_) [3]. In this study, *d_0_* was set to 5.3 mm, based on the dimensions of the mold used for hydrogel preparation.

### 2.11. Compression testing

Hydrogels were put on compression tester. Compression tests were conducted at a constant speed of 1 mm/min. The applied force (*N*) was converted into compressive stress (*G*′) using the Neo-Hookean model. The surface area of each hydrogel sample were measured by optical microscopy prior to testing to ensure accurate stress calculation. Additionally, the height of each hydrogel was calculated from the as-prepared height and the swelling ratio.

### 2.12. SEM observation

PEG sponge hydrogels (10 and 60 g/L) were sectioned using an ultrasonic cutter and then flash-frozen in liquid nitrogen at −196°C. After freeze-drying for 24 h, the dried sections were mounted on SEM stubs with carbon tape and sputter-coated with osmium. Surface and cross-sectional images – both the outer and inner regions – were acquired on a JEOL JSM-7500FA scanning electron microscope.

### 2.13. Liesegang ring observation

Hydrogels were cast in silicone molds (30 mm × 10 mm × 3 mm). After gelation, the samples were immersed in K300 for 24 h, rinsed with Tris-HCl buffer, and then placed between two glass plates. The C500 was introduced into the space between the plates, ensuring that it did not contact the hydrogel surfaces adjacent to the glass. To investigate the pattern formation, the spacing coefficient (*p*) was calculated from the distribution of liesegang bands formed in different hydrogels. The coefficient *p* was determined using the following equation:

$$x_{n+1}=f\left(x_{n}\right)=\frac{1}{1+p}x_{n}$$

where $x_{n}$ is the distance of the *n*_th_ band from the edge. A larger *p* value indicates greater spacing between successive bands and is interpreted as enhanced nucleation activity of HAp precipitates.

## 3. Results and discussions

### 3.1. Simple preparation of HAp-deposited PEG sponge hydrogel

Efficient mineralization of synthetic hydrogels requires sufficient ionic transport through the network while maintaining structural integrity. To this end, we employed PEG sponge hydrogel fabricated through GGPS followed by freeze – thaw processing [20]. The resulting hydrogel consists entirely of PEG at a low polymer concentration (10 g/L), forming a three-dimensional network with ~99% water content and micrometer-scale interconnected pores. This architecture contrasts sharply with conventional PEG hydrogels, which typically feature dense, nanometer-scale meshes that hinder mass transport. We hypothesized that the open-channel morphology of the PEG sponge hydrogel would enhance ion diffusion and support deep HAp deposition throughout the hydrogel bulk, provided the mineralization protocol was suitably optimized. As a first benchmark, we applied a standard alternating immersion technique – repeated sequential exposure to K_2_HPO_4_ and CaCl_2_ solutions – previously used for HAp formation in hydrogel systems [22]. However, when applied to the PEG sponge hydrogel, this approach led to extensive surface cracking and structural deformation, with mineralization restricted to the outermost regions (Figure 1(B)). These failures underscore the incompatibility of traditional protocols with soft, low-modulus hydrogels, where repeated osmotic shocks exceed the mechanical limits of the network and compromise both form and function.

To circumvent these issues, we developed a simplified immersion protocol using stoichiometrically optimized phosphate and calcium salt solutions (Figure 1(A)). Unlike alternating methods that cycle between ion sources [23], this ‘simple immersion’ approach involves sequential immersion in each ion solution under tightly controlled conditions, minimizing osmotic imbalance and promoting nucleation within hydrogels. Remarkably, this strategy yielded uniform, macroscopically white hydrogels indicative of extensive mineralization. Cross-sectional imaging confirmed mineral deposition extending over 1 mm into the interior – substantially deeper than previously reported for low-polymer-content PEG hydrogels using more complex procedures [10]. These findings demonstrate that the porous structure of the PEG sponge hydrogel is not merely a passive scaffold but a critical enabler of deep bulk mineralization. By enhancing mass permeability and maintaining sufficient PEG chain density for Ca^2+^ coordination, the network supports in situ HAp formation under mild conditions. Importantly, the system requires no porogens, biopolymer additives, or external stimuli, highlighting its compositional simplicity and scalability for bioinspired mineral – hydrogel composites.

### 3.2. Structural characterization of PEG sponge hydrogel

**Figure 2.**
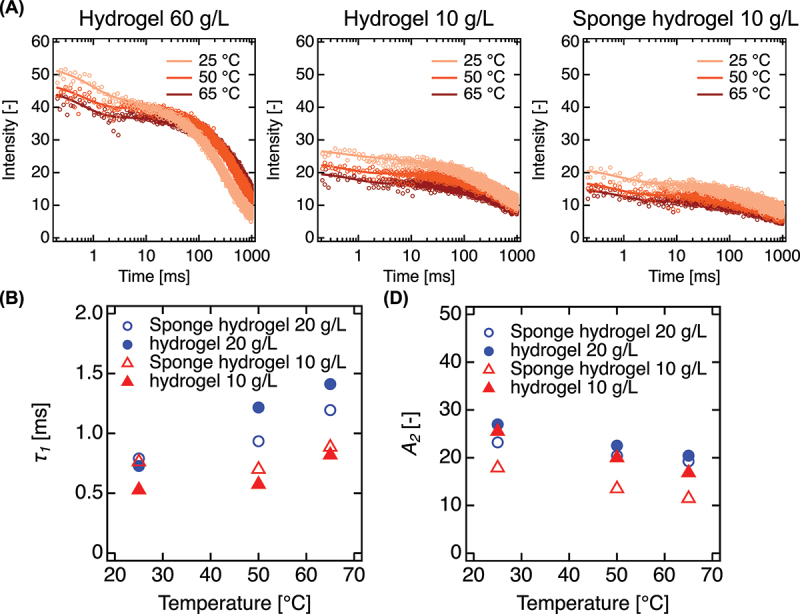
Structural characterization of PEG sponge hydrogels. (A) Representative ^1^H NMR echo signal decay curves for conventional PEG hydrogels (10 g/L and 60 g/L) and PEG sponge hydrogels (10 g/L) measured at 25, 50, and 60°C. Decay curves were fitted using a two-component exponential model to extract fast and slow relaxation components. (B) Temperature-dependent behavior of the fast and slow-relaxing component, shown as relaxation time (*τ_1_*) and *A_2_*.

To elucidate the structural features that enable deep mineralization in PEG sponge hydrogels, we examined the internal polymer dynamics (Figure 2). The PEG sponge hydrogel consists of dense PEG-rich domains embedded in a highly hydrated matrix – a microarchitecture generated by GGPS and modulated by polymer concentration. As the polymer concentration decreases, the hydrogel exhibits increasingly pronounced porosity, characterized by sparsely distributed but highly interconnected PEG domains (Figure S1). To assess molecular mobility within these networks, we employed ^1^H NMR relaxation measurements using Carr – Purcell – Meiboom – Gill (CPMG) and spin echo pulse sequences[23] (Figure 2(A)). All samples showed biphasic relaxation profiles, indicating two distinct proton populations. The slower-relaxing component arises from residual water molecules retained after D_2_O exchange, reflecting highly mobile environments. In contrast, the faster-relaxing component originates from PEG protons under structural constraints, providing insight into local network rigidity. The relative amplitude of each component was extracted from two-component exponential fits and denoted as *A_2_*, representing the fraction of constrained PEG chains. Hydrogels with high polymer content exhibited strong echo signal intensities and increasing *τ_1_* values with rising temperature, indicating enhanced segmental mobility of PEG chains at elevated thermal energy. As the polymer concentration decreased, the temperature dependence of *τ_1_* diminished, consistent with the emergence of immobilized microdomains (Figure 2(B)). Notably, PEG sponge hydrogels (10–20 g/L) showed elevated *τ_1_* values and significantly reduced temperature sensitivity – indicating that the PEG chains were confined within rigid, aggregated domains (Figure 2(B)). This behavior mirrors that of systems with thermally stabilized polymer aggregates, where chain mobility is restricted despite low bulk concentration. Furthermore, the concurrent increase in *τ*_*1*_ and decrease in *A*_*2*_ with rising temperature implies the progressive aggregation of PEG chains. These findings establish that freeze – thaw-induced PEG sponge formation results in a paradoxical state: low polymer density coupled with locally rigid domains. This combination yields two critical features for mineralization. First, the interconnected porous structure permits high ionic diffusivity, overcoming the transport limitations of conventional hydrogels. Second, the dense PEG-rich microdomains offer abundant sites for Ca^2+^ coordination and heterogeneous nucleation. Together, these structural and dynamic characteristics create a favorable environment for deep HAp deposition. Unlike standard PEG hydrogels, which typically confine mineralization to surface regions due to restricted ion mobility and sparse nucleation, PEG sponge hydrogels support mineral growth across the entire bulk matrix.

### 3.3. Optimization of hydrogel mineralization protocols

To develop an effective mineralization strategy for PEG sponge hydrogels, we first examined previously reported alternating immersion protocols using phosphate (K_2_HPO_4_, 300 mM) and calcium (CaCl_2_, 500 mM) solutions (2 min each). However, when these conditions were applied to PEG sponge hydrogels, uneven mineral deposition, insufficient penetration into the hydrogel interior, and pronounced structural damage were observed upon repeated cycling (Figure S2). These results indicate that short immersion times are inadequate for ion diffusion in highly hydrated, low-modulus sponge hydrogels and that repeated ionic shocks destabilize the network. Based on these observations, we concluded that achieving HAp deposition within a single mineralization cycle is essential for maintaining hydrogel integrity while enabling deep mineralization. We therefore optimized each immersion step independently, as described below.

**Figure 3.**
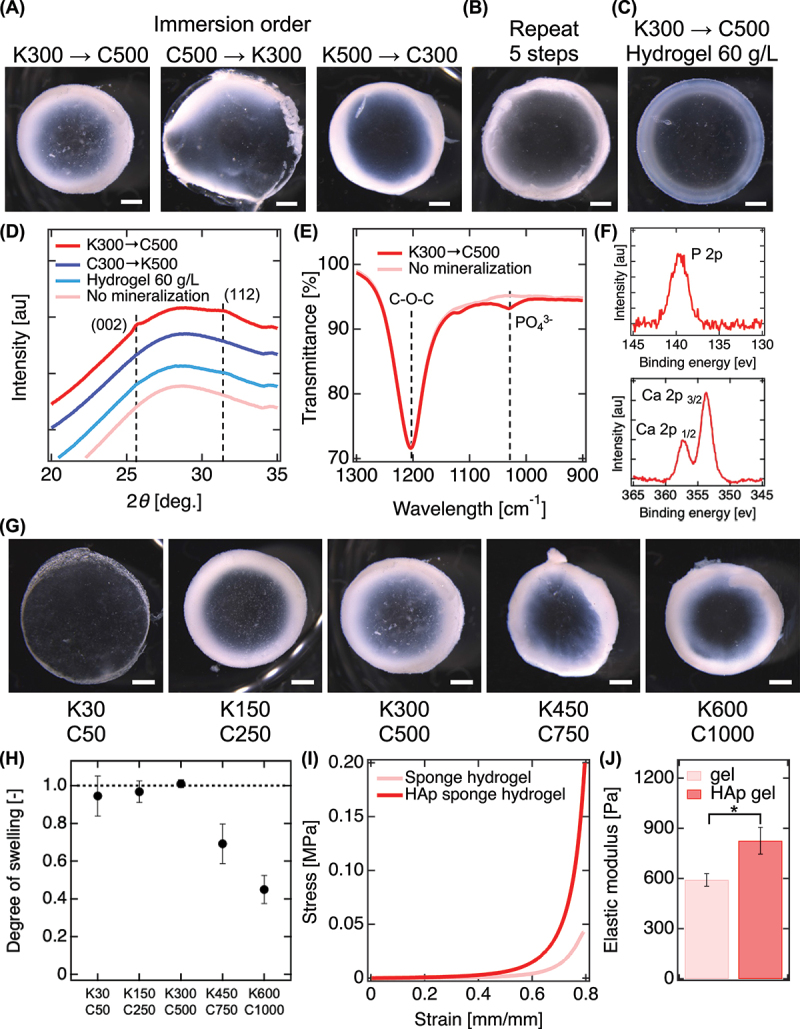
Optimization of hydrogel mineralization protocols. (A) Cross-sectional images of PEG sponge hydrogels (10 g/L) subjected to simple immersion protocols. Samples were immersed in K_2_HPO_4_ (K) and CaCl_2_ (C) solutions with varying sequences and concentrations. (B) Cross-sectional images of PEG sponge (10 g/L) following five cycles of alternating immersion in K300 and C500. (C) Cross-sectional images of dense PEG hydrogels (60 g/L) mineralized via simple protocols. (D) 1D WAXS profiles of hydrogels after mineralization under different conditions. Dashed lines mark the characteristic hydroxyapatite reflections at 2θ = 26° (002) and 32° (112). (E) FT-IR spectra of PEG sponge (10 g/L) after simple mineralization. Dashed lines indicate characteristic peaks of C–O–C stretching (~1200 cm^−1^) and phosphate vibrations (~1020 cm^−1^). (F) XPS spectra of mineralized sponge hydrogels showing P 2p (130–145 eV) and Ca 2p (345–365 eV) binding energy regions. (G) Cross-sectional images of sponge hydrogels mineralized under different total salt concentrations. (H) Equilibrium degree of swelling (*Q*) for sponge hydrogels mineralized under varying conditions. Dashed line at *Q* = 1.0 represents the transition between swelling and shrinkage. (I) Compressive stress – strain curves for mineralized hydrogels. (J) Calculated elastic modulus derived from compressive stress – strain profiles. Scale bars: 1 mm for all cross-sectional images. Data are presented as mean ± standard deviation, *n* = 3. *p < 0.05.

Leveraging the enhanced mass permeability of PEG sponge hydrogels, we developed a simplified sequential immersion protocol for HAp mineralization (Figure 3). Solution conditions were designated as Kx and Cx, corresponding to immersion in K_2_HPO_4_ or CaCl_2_ at concentration ×(mM). Among all tested conditions, sequential immersion in K300 followed by C500 yielded macroscopically uniform, opaque-white hydrogels, indicative of continuous and deep mineral formation throughout the matrix (Figure 3(A)). In contrast, reversing the immersion order (C500→K300) or deviating from the ideal Ca/P molar ratio (1.67) (K500→C300) resulted in inhomogeneous mineral distribution, surface cracking, or hydrogel collapse. These outcomes are likely attributable to phosphate-induced PEG dehydration, which destabilizes the hydrogel network and impedes nucleation prior to calcium-mediated stabilization. Only the K300→C500 condition under the correct Ca/P ratio produced diffraction peaks at 2θ = 26° and 32° in wide-angle X-ray scattering (WAXS), corresponding to the (002) and (112) reflections of crystalline HAp, in agreement with standard reference data (ICSD 137,671) (Figure 3(D)). It should be noted that absolute crystallinity cannot be reliably quantified from WAXS in this hydrogel-based system due to the amorphous contribution of the PEG matrix. Instead, the crystal quality was evaluated by comparing the sharpness and relative prominence of the characteristic HAp reflections at 2θ ≈ 26° (002) and 32° (112) (Figure S3). Under the optimized conditions, these reflections were clearly resolved, whereas they were weak or absent under non-ideal conditions, indicating less ordered mineral phases. These observations highlight the critical role of both immersion order and stoichiometric control in directing nucleation and phase development. Notably, repeating the immersion cycle five times – conventional protocols – resulted in substantial hydrogel shrinkage and visible deformation, highlighting the mechanical advantage of the sequential protocol over traditional methods (Figure 3(B)). Furthermore, the efficiency of mineralization was highly dependent on hydrogel architecture (Figure 3(C)). Dense, non-porous PEG hydrogels prepared at 60 g/L exhibited only peripheral mineralization and no detectable HAp peaks in WAXS, confirming limited ionic penetration. In contrast, PEG sponge hydrogels displayed phosphate vibrational bands (~1050 cm^− 1^) in FT-IR spectra and distinct Ca 2p and P 2p signals in XPS (Figure 3(E,F)), validating the formation of HAp in the hydrogel [16,24]. Collectively, these results demonstrate that the combination of interconnected porosity and a single-cycle sequential immersion protocol enables structurally robust and spatially deep mineralization. By avoiding repeated ionic shock and diffusion-limited surface precipitation, this approach overcomes the limitations associated with conventional alternating protocols and provides a reliable strategy for engineering mineralized hydrogels with controlled architecture and composition.

### 3.4. Salt concentration effect on hydrogel mineralization

Following optimization of the immersion sequence and ionic stoichiometry, we next investigated the influence of salt concentration on HAp deposition and hydrogel structural stability. While elevated ionic strength enhances ion flux and promotes nucleation [25], excessive concentrations can induce osmotic stress and phase destabilization in soft hydrogels, compromising mechanical integrity. To identify a concentration regime that supports both efficient mineralization and network preservation, we systematically varied the molarities of K_2_HPO_4_ and CaCl_2_ while maintaining a fixed Ca/P ratio of 1.67, and quantified the equilibrium swelling ratio (*Q*) after mineralization as a metric of structural retention (Figure 3(G,H)). At high ionic concentrations (e.g. K450 and C750), hydrogels exhibited severe shrinkage (*Q * < 1), indicating substantial dehydration and potential phase collapse during mineral infiltration. Although these conditions yielded extensive HAp deposition, the resulting hydrogels suffered from pronounced deformation and poor mechanical usability. In contrast, a moderate concentration condition (K300 and C500) produced deeply mineralized hydrogels that retained their macroscopic form with minimal swelling-induced distortion. This balance of crystallization and structural integrity suggests an optimal window for salt-mediated mineralization. Mechanical testing confirmed that mineralization under the optimized conditions enhanced compressive modulus and yield strength (Figure 3(I,J)), attributed to in situ HAp crystallization reinforcing the PEG network. Mechanical properties were systematically evaluated for all hydrogel compositions and mineralization conditions presented in this study, including different polymer concentrations and salt conditions (Figures S4, S5). Compared to traditional alternating immersion protocols, the streamlined sequential method not only simplified the process but also delivered comparable or enhanced mechanical reinforcement without inducing network failure. These results highlight the importance of ionic strength regulation in hydrogel mineralization. By maintaining chemical stoichiometry while optimizing salt concentrations, the protocol achieves deep mineral penetration and mechanical enhancement, all while preserving the hydrogel’s soft, porous architecture – key features for functional scaffold design.

Following optimization of the mineralization conditions, we examined whether the porous architecture of the PEG sponge hydrogels was preserved after HAp deposition. CLSM combined with image-based analysis revealed that the overall porosity, particularly in the 10 g/L sponge hydrogels, remained largely unchanged after mineralization (Figure S1). This indicates that HAp formation occurs predominantly along the internal pore walls rather than through complete pore filling. The preservation of interconnected porosity is critical, as it maintains continuous diffusion pathways within the hydrogel even after mineralization. This structural retention provides a mechanistic explanation for the observed deep mineral infiltration and distinguishes the present system from conventional approaches, in which surface-localized deposition often leads to pore blockage and structural collapse.

### 3.5. Effect of porous structure of PEG sponge

**Figure 4.**
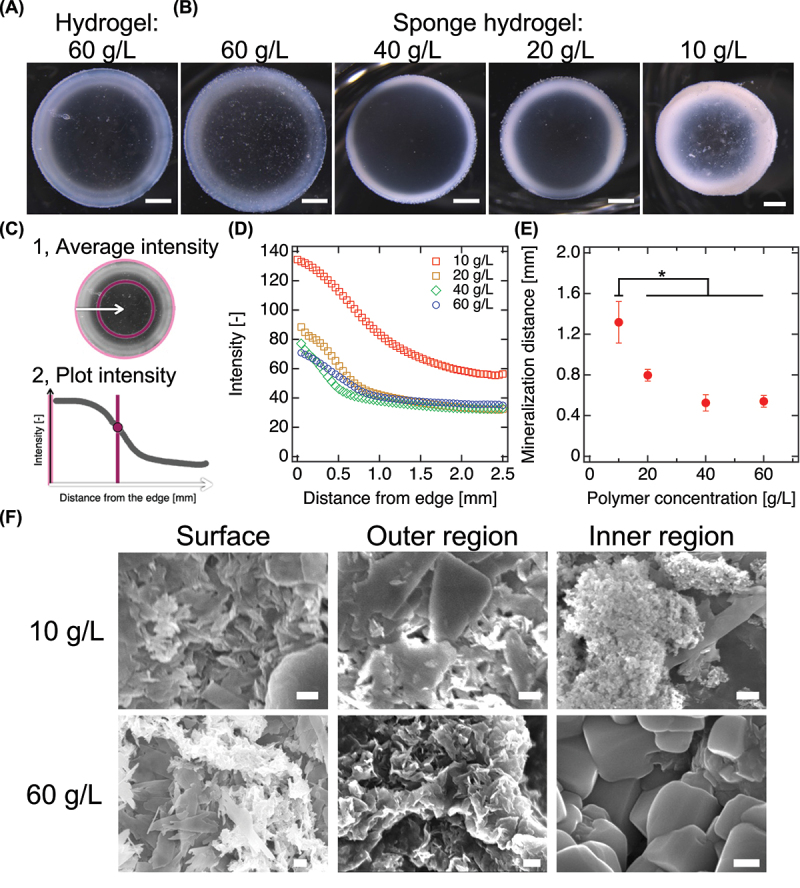
Effect of hydrogel architecture on mineralization depth and morphology. (A, B) Cross-sectional optical images of mineralized Tetra-PEG hydrogels (60 g/L) (A) and PEG sponge hydrogels (10–60 g/L) (B) prepared under simple immersion conditions. Scale bars, 1 mm. (C) Schematic illustration of the image analysis protocol used to quantify mineral penetration. Radial whiteness intensity was computed by measuring the annular mean brightness from the hydrogel periphery toward the center. (D) Plots of annular mean whiteness intensity as a function of radial distance from the hydrogel edge. Image analyses were performed using ImageJ. (E) Quantified mineralization distance. Data are presented as mean ± standard deviation (n = 3). *p* < 0.05. (F) SEM images of the hydrogel surface and interior following mineralization. Scale bars, 200 nm.

To elucidate the role of internal architecture in modulating hydrogel mineralization, we systematically varied the polymer concentration (10–60 g/L) in PEG sponge hydrogels and applied the optimized sequential immersion protocol (Figure 4(A,B)). While all samples exhibited visible peripheral whitening, cross-sectional analysis revealed pronounced differences in mineral penetration and spatial uniformity depending on morphology. Quantitative image analysis was performed by extracting radial brightness profiles from concentric cross-sections, allowing precise assessment of mineral distribution (Figure 4(C)). Low-polymer hydrogels (notably the 10 g/L sponge hydrogel) exhibited markedly greater mineral infiltration, with high whiteness intensity extending deep into the core (Figure 4(D,E)). These hydrogels achieved approximately double the mineralized distance compared to their high-concentration (60 g/L) counterparts, demonstrating that porosity – rather than polymer mass – dictates mineral accessibility. This enhanced mineralization is attributed to the freeze – thaw-induced microarchitecture of low-concentration PEG sponge hydrogels. The resulting micron-scale, interconnected pore network promotes efficient electrolyte diffusion and increases the surface availability of PEG chains for Ca^2+^ coordination – critical factors for nucleation and crystal propagation. Scanning electron microscopy (SEM) supported this interpretation: while surface regions and outer areas of the 60 g/L sponge hydrogels exhibited fine, flake-like HAp crystals, the interior contained markedly larger crystalline domains (Figure 4(F)). In contrast, the 10 g/L PEG sponge hydrogels showed densely dispersed nanocrystals throughout the matrix. This spatial pattern is consistent with the notion that limited ion diffusion in dense hydrogels leads to ion accumulation in the core, creating locally supersaturated environments that favor the formation of fewer, but larger, crystals [26]. Conversely, the porous architecture of low-polymer hydrogels facilitates widespread ion transport and distributed nucleation, resulting in finer mineralization. These findings demonstrate that hydrogel porosity is a key design parameter for enabling deep spatially mineral deposition. The structural transition from nanometric mesh to sponge-like architecture enhances both permeability and functional ion-binding capacity, thereby unlocking new opportunities for engineering bioinspired materials. Such architecture-guided mineralization provides a platform for developing osteoconductive scaffolds and templated crystallization systems with good structural integration and functional tunability.

Importantly, while pore size is a dominant factor governing mineralization behavior, the present system does not allow a strict one-to-one quantitative correlation between pore size and mineralization efficiency. Mineral deposition in hydrated PEG networks is influenced not only by geometric pore dimensions but also by coupled factors such as network density, diffusional tortuosity, elastic deformation during ion uptake, and the kinetics of hydroxyapatite nucleation and growth. Nevertheless, a clear semi-quantitative trend is evident: hydrogels with larger, more accessible pore networks (e.g. 10 g/L PEG sponge) consistently exhibit deeper and more homogeneous mineralization, whereas denser hydrogels with smaller pores restrict ion transport and limit mineral penetration. This correlation demonstrates that pore accessibility, rather than pore size alone, serves as a key structural determinant of mineralization efficiency in PEG sponge hydrogels.

### 3.6. Mechanistic insights from Liesegang ring formation

**Figure 5.**
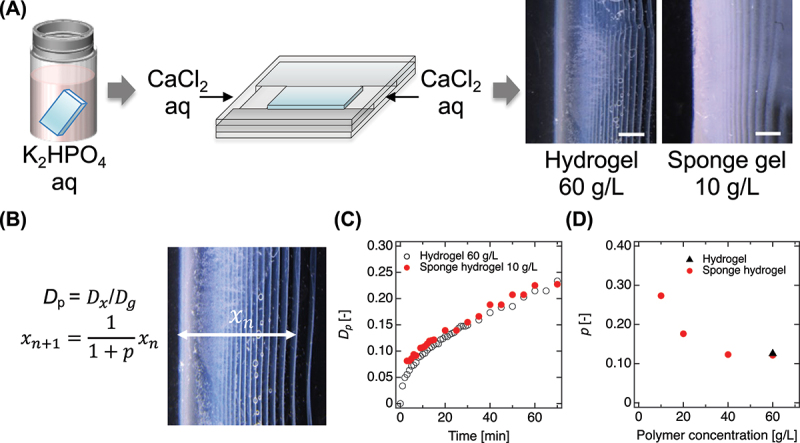
Liesegang ring formation in hydrogels. (A) Schematic of the experimental setup to evaluate Liesegang ring formation using the simple mineralization protocol. PEG-based hydrogels were preloaded with phosphate and exposed to a calcium source, initiating reaction – diffusion-driven precipitation. Inset: cross-sectional images of Tetra-PEG (right) and PEG sponge (left) hydrogels after mineralization, showing distinct banding patterns. Scale bars, 1 mm. (B) Analytical framework used to quantify precipitation behavior. The degree of penetration (*D*_p_) was defined as the ratio of precipitation front distance (*D*_x_) to the total hydrogel length (*D*_g_). The spacing coefficient (*p*) was calculated from the relative positions of successive Liesegang bands (*x*_n_). (C) Temporal evolution of *D*_p_ for PEG sponge and Tetra-PEG hydrogels. (D) Correlation between polymer volume fraction and the spacing coefficient (*p*).

To uncover the mechanism enabling deep HAp deposition in PEG sponge hydrogels, we examined the precipitation pattern dynamics during mineralization, focusing on the emergence of Liesegang rings – periodic structures characteristic of reaction – diffusion-driven crystallization. Liesegang rings result from nonlinear electrolyte diffusion and local supersaturation, producing self-organized precipitation bands that emerge as mobile ions (e.g. Ca^2+^) infiltrate a matrix preloaded with counter-ions (e.g. phosphate) [16–18,26]. This reaction front induces alternating zones of nucleation and depletion, and has been widely reported in mineralized hydrogels. We investigated Liesegang behavior in structurally distinct PEG hydrogels: conventional dense Tetra-PEG networks and porous PEG sponge architectures (Figure 5). Both systems showed initial HAp formation at the interface with CaCl_2_ solution, indicating comparable early-stage nucleation kinetics despite their contrasting network structures (Figure 5(C)). However, their spatial pattern evolution diverged substantially. While conventional hydrogels exhibited faint or incomplete banding limited to the near-surface regions, PEG sponge hydrogels developed clear Liesegang rings that extended deeply into the matrix. This pronounced periodicity suggests that the sponge architecture supports sustained reaction front propagation and long-range nucleation. To quantify this effect, we calculated the Liesegang spacing coefficient (*p*), which describes the relative spacing between successive precipitation bands and reflects the nonlinearity of the diffusion front (Figure 5(D)). Higher *p* values correspond to steeper ion gradients, stronger supersaturation, and greater mineral accumulation [27]. PEG sponge hydrogels exhibited significantly elevated *p* values compared to standard PEG hydrogels, confirming their enhanced capacity to support volumetric, periodic mineralization. These behaviors are attributed to the sponge’s unique structural features: high ionic permeability, and extensive internal surface area for ion coordination and nucleation. Together, these attributes enable robust bidirectional diffusion of calcium and phosphate ions, prevent early depletion of reactants, and suppress termination of the Liesegang reaction front. In contrast, dense hydrogels rapidly reach ion-depleted states that limit band formation and confine mineralization to peripheral regions.

These results provide critical mechanistic insight into the advantages of sponge hydrogel architectures for solution-based crystallization. By maintaining favorable ion transport conditions and sustaining nucleation fronts, the PEG sponge hydrogel overcomes classical transport-limited constraints of Liesegang patterning. This strategy opens a new avenue for mineralization in hydrogels, enabling spatially controlled, volumetric crystal growth that was previously inaccessible in soft materials.

## 4. Conclusion

This study establishes a robust and scalable strategy for deep HAp mineralization in PEG hydrogels by coupling a sponge-like porous network with an optimized immersion protocol. Engineered via GGPS and cryogenic structuring, the PEG sponge hydrogel exhibits a unique combination of high water content, low modulus, and structurally constrained polymer domains – features that collectively promote efficient bidirectional ion transport and spatially extensive mineral nucleation. Unlike conventional hydrogels limited by surface-localized Liesegang ring formation, this architecture enables continuous HAp deposition across millimeter-scale depths without structural degradation. Beyond demonstrating a simple and additive-free method for mineralized hydrogel fabrication, our findings reveal generalizable design principles for overcoming transport barriers in soft matter systems. By tuning porosity, polymer dynamics, and ionic conditions, this platform opens new avenues for constructing bioinspired soft – mineral composites, with significant implications for bone-mimetic scaffold development and regenerative material design.

## Supplementary Material

Supplemental Material
