# Supplementary material for: Deep hydroxyapatite deposition in porous poly(ethylene glycol) sponge hydrogel via optimized and simplified approach
Source: Sci Technol Adv Mater. 2026 Jan 23;27(1):2620828. doi: 10.1080/14686996.2026.2620828 (PMC12943808; doi:10.1080/14686996.2026.2620828)
Supplement: Supplemental Material [file TSTA_A_2620828_SM0906.docx]

**Deep hydroxyapatite deposition in porous poly(ethylene glycol) sponge hydrogel via optimized and simplified approach**

Kaho Takada^1^, Shohei Ishikawa^1,*^, Rikima Kuwada^1^, Lester Geonzon^2^, Koichi Mayumi^2^, Takamasa Sakai^1,*^

[1] Department of Chemistry & Biotechnology, School of Engineering, The University of Tokyo, 7-3-1 Hongo, Bunkyo-ku, Tokyo, 113-8656, Japan

[2] Institute for Solid State Physics, The University of Tokyo, 5-1-5 Kashiwanoha, Kashiwa, Chiba, 277-8581, Japan

*Corresponding Authors:

S.I. ([Ishikawa@gel.t.u-tokyo.ac.jp](mailto:Ishikawa@gel.t.u-tokyo.ac.jp))

T.S. ([sakai@gel.t.u-tokyo.ac.jp](mailto:sakai@gel.t.u-tokyo.ac.jp))

**Key words**

Hydrogel, Hydroxyapatite, mineralization, poly(ethylene glycol)

**Figure S1.** (**A**) Confocal laser scanning microscopy (CLSM) images of PEG sponge hydrogels with and without HAp deposition (HAp(+) and HAp(−)). Scale bars: 100 μm. (**B, C**) Porosity (**B**) and condensation ratio (**C**) of PEG sponge hydrogels.

**Figure S2**. Cross-sectional images of PEG sponge (10 g/L) following five cycles of alternating immersion in K300 and C500. Scale bars, 1 mm.

**Figure S3.** 1D WAXS profiles of hydrogels after mineralization under different salt conditions. Dashed lines mark the characteristic hydroxyapatite reflections at 2θ = 26° (002) and 32° (112).

**Figure S4.** Calculated elastic modulus of hydrogels with various polymer concentration.

**Figure S5.** Calculated elastic modulus of hydrogels with various salt concentration.
